# Know your enemy: Unexpected, pervasive and persistent viral and bacterial contamination of primary cell cultures

**DOI:** 10.1111/exd.14126

**Published:** 2020-06-25

**Authors:** Hanna Niehues, Patrick A. M. Jansen, Diana Rodijk‐Olthuis, Gijs Rikken, Jos P. H. Smits, Joost Schalkwijk, Patrick L. J. M. Zeeuwen, Ellen H. J. van den Bogaard

**Affiliations:** ^1^ Department of Dermatology Radboud University Medical Center (Radboudumc) Radboud Institute for Molecular Life Sciences (RIMLS) Nijmegen The Netherlands

**Keywords:** adenovirus, antibiotics, cell culture, infection, keratinocyte, spore‐forming bacteria

## Abstract

In biomedical research, cell culture contamination is one of the main culprits of experimental failure. Contamination sources and concomitant remedies are numerous and challenging to manage. We herein describe two cases of uncommon contamination of cell cultures that we encountered, and the successful determination and eradication strategies. The first case describes the infection with human adenovirus C that originated from pharyngeal tonsils used for isolation of primary tonsillar epithelial cells. It is known that viral contamination of in vitro cell cultures can occur symptomless and is therefore difficult to identify. The contamination was pervasive and persistent, as it was widely spread in flow cabinets and apparatus, and has caused a serious delay to our research projects and the inevitable loss of valuable (patient‐derived) cell sources. Eradication was successful by formalin gas sterilization of the flow cabinet and elimination of all infected cell lines from our biobank after PCR‐guided determination. Secondly, we encountered a spore‐forming bacterium, namely *Brevibacillus brevis,* in our cell culture facility. This bacterium originated from contaminated tap water pipes and spread via regular aseptic culture techniques due to survival of the bacterial spores in 70% ethanol. *B brevis* overgrew the cultures within a few days after seeding of the primary cells. Chlorine solution effectively killed this spore‐forming bacterium. Both cases of contamination were identified using DNA sequencing which enabled the deployment of targeted aseptic techniques for the elimination of the persistent contamination.

**Abbreviations**
HAdV: human adenovirus
LCE: late cornified envelope
pen/strep: penicillin/streptomycin

## 1 BACKGROUND

Cell culture models are a gold standard in biomedical research to mimic human cell‐cell and cell‐tissue interactions.^[^ ^1^, ^2^ ^]^ For conventional monolayer and state‐of‐the‐art organotypic tissue cultures, primary cells can be isolated from various human tissues. Experimental failure of cell isolation procedures is not only time‐consuming and costly, but high success rates of primary cell cultures are demanded due to the limited availability of human tissue. Contamination is a major threat for research involving cell cultures, and especially for organotypic tissue cultures given the extensive culture periods, valuable cellular resources and substantial costs for defined media and specialized cell culture equipment.

The main cell culture contaminants are bacteria, fungi and yeasts, and a substantial proportion of all commonly used cell lines were found to be contaminated by the intracellular *Mycoplasma* bacterium.^[^ ^3^, ^4^ ^]^ Most bacterial and fungal infections are introduced by inadequate aseptic culture techniques. In general, working in a sterile flow hood combined by cleaning work surfaces and consumables with 70% ethanol will protect against cell culture contamination. Ethanol penetrates cell walls and denatures protein and lipid structures, and however, some bacteria, fungi and viruses appear insensitive to ethanol.^[^ ^5^ ^]^ To successfully combat cell culture contamination, it is therefore of utmost importance to know your enemy and choose the right aseptic mode of defence.

To reduce the risk of contamination, cell culture facilities worldwide supplement culture medium with broad‐spectrum antibiotics (eg penicillin/streptomycin, gentamicin, kanamycin, neomycin) and antimycotics (eg amphotericin B, nystatin).^[^ ^6^ ^]^ However, the effect of prophylactic use of antibiotics should not be underestimated. We, and others, have shown that the use of antibiotics can hamper cellular responses.^[^ ^7^, ^8^, ^9^, ^10^ ^]^ Additionally, non‐stop prophylactic use of antibiotics may lead to bacterial resistance.

Therefore, we adopted antibiotic‐free cell culture models a few years ago, but also learned that several contamination sources should be taken into account and that the identification of contamination is vital for proper decontamination. From our experiences, we herein describe two cases of putatively uncommon, yet extremely persistent sources of cell culture contamination, namely tonsillar‐derived human adenovirus (HAdV) C and a spore‐forming *Brevibacillus* sp bacterium which originated from laboratory tap water systems.

## 2 QUESTIONS ADDRESSED

We herein address the strategy developed to identify and eradicate cell culture contaminants of extraordinary origin.

## 3 EXPERIMENTAL DESIGN

The methods for primary human keratinocyte cultures, bacterial growth on blood agar plates, microbial DNA isolation, PCR amplification, sequencing and HAdV C‐specific qPCR are described in detail in Data S1.

## 4 RESULTS

### 4.1 Case 1: HAdV C

**FIGURE 1:**
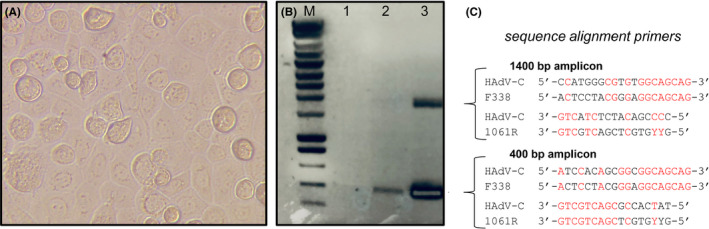
HAdV C infection of keratinocyte cultures. A, Keratinocyte monolayer culture showing cells with black spots (arrows), enlarged cells (asterisk) and flattened rounded cells (arrowheads) indicating cell death. B, Agarose gel electrophoresis of 16S rRNA PCR amplicons of keratinocytes isolated prior to HAdV C contamination (*lane 1, no band*) and two different HAdV C infected keratinocyte cultures (*lane 2‐3*). M = 200 bp Smart ladder (Eurogentec). C, Universal 16S rRNA F338/1061R primers fit partly with 3’‐nucleotides (in red) on HAdV C sequence

The major activity of our laboratory is to study the biology of epidermal keratinocytes using primary cells. For comparison with oral epithelia, we aimed to establish primary cells from mouth and throat tissues. After we commenced the collection and processing of pharyngeal tonsils for isolation of primary tonsillar epithelial cells, we encountered serious problems with all our other keratinocyte cultures a few weeks later. Submerged cultures of primary keratinocytes as well as immortalized N/TERT keratinocytes reached a confluency of maximal 30%‐40%, but 5 thereafter all cells died within 5‐7 days. Cells showed suspicious blebs on the colonies and/or black spots in the cells (Figure 1A). *Mycoplasma* tests were negative. Next, we collected supernatants from affected keratinocyte cultures for plating on blood agar as a basic test for bacterial contamination. Overnight aerobic and anaerobic culture at 37°C did not reveal bacterial growth on the blood agar plates, nor after three days of incubation. To exclude detrimental effects due to sub‐optimal cell culture conditions, we tested different batches of culture media, supplements, culture plastics etcetera but without conclusive results. Knowing that only a small part of all microorganisms can be grown under laboratory conditions,^[^ ^11^ ^]^ we hypothesized that, although the initial blood agar tests were negative, bacterial infections might be to blame. To test this hypothesis, we isolated genomic DNA from the infected keratinocyte cultures and used this as a template for a PCR reaction amplifying the V3‐V6 region of the 16S rRNA gene using universal F338/1061R primers.^[^ ^12^ ^]^ With agarose gel electrophoresis, we did not find the expected amplicon with a size of 750 bp but instead two unexpected bands of approximately 400 and 1400 bp, respectively (Figure 1B). Both bands were purified and subsequently sequenced. Surprisingly, Blast searches with sequences from both bands revealed that these encoded human adenovirus C (HAdV C). Sequence analysis indicated that both amplicons could indeed be generated due to a partial match of the F338/1061R primers with the HAdV C sequence at the 3' end (Figure 1C). Specific HAdV C qPCR primers enabled a rapid detection test for our cell bank and complete batches of infected cells were discarded. The HAdV C contamination was found to circulate in the laminar flow cabinets and appeared to be infectious for a prolonged time, leading to repeated infections even after standard cleaning of cabinets and apparatus. HAdV C was successfully eradicated by formalin gas sterilization of our entire cell culture facility.

### 4.2 Case 2: *Brevibacillus* sp

About one year after the HAdV C contamination, we encountered a second (unrelated to the HAdV C) extraordinary contamination. We observed that a few days following seeding of primary keratinocytes the media of these cultures showed clouding, indicating a bacterial infection, in absence of the acidification of the culture medium. Again, cultures were negative for *Mycoplasma*. The supernatant of the affected cultures was applied to blood agar plates where white/grey bacterial colonies appeared after overnight aerobic culture at 37°C. Microscopic preparations showed rod‐shaped bacteria, suggestive of a bacillus. Unexpectedly, after thorough disinfection of all equipment using standard 70% ethanol, and the use of proven sterile media, the newly started cultures were again found to be infected. To identify its nature, we performed a PCR to amplify the V3‐V6 region of the 16S rRNA gene of this microorganism, as described in Case 1 above. The identity of the isolate was determined as *Brevibacillus* sp based on partial 16S rRNA sequence data with *B brevis* as the closest species.^[^ ^13^ ^]^ *Brevibacillus* sp are gram‐positive, aerobic, spore‐forming bacteria resistant to ethanol.^[^ ^14^ ^]^ Given this knowledge, we searched for the infection source. Turbidity of the water baths directed us to take samples of all water taps, which all proved positive for the same white/grey colonies we found in our cell cultures. Regular used chemical biocides against spore‐forming bacteria are chlorine‐based detergents.^[^ ^15^ ^]^ Therefore, we treated water samples with a low concentration of chlorine (50 mg/L, pH 7.0), which effectively killed the spores and prevented germination (Figure S1). Because the highest concentration of bacteria was found in the demineralized water tap, we were able to trace the infection to the ion exchanger used for generation of demin water. The bacterial contamination was successfully eliminated by chlorine treatment of the water pipe system and replacement of the ion exchanger cartridge. Following these procedures, and cleaning of laminar flow cabinets and apparatus with chlorine, no more contaminations of the cell cultures with this bacterium were encountered.

## 5 CONCLUSIONS

We faced rare but persistent cell culture contaminants which significantly delayed our research projects and required “blood, sweat and tears” to eliminate. In the hope of helping others with similar issues, we believe it is of utmost important to share our experiences and methodologies in tackling the contaminations we encountered. In this report, we describe the identification and elimination strategies for two cell culture contaminations. Although we encountered them primarily in our human keratinocyte cultures, other cell types were equally affected (eg mouse fibroblasts (3T3‐J2) and human embryonic kidney cells (HEK293T)). Hence, it is of importance to share our experiences with the scientific commmunity.

The introduction of HAdV C in our cell culture facility was an unfortunate consequence of our efforts to generate 3D reconstructed oral epithelia such as gingiva, palatum and tonsil. For the isolation of tonsillar epithelial cells, we used primary tissue from pharyngeal tonsils of children who frequently suffered from tonsillitis leading to tonsillectomy. Unfortunately, recurring tonsillitis is most frequently associated with HAdV^[^ ^16^ ^]^ and also reported since the early 1950s.^[^ ^17^, ^18^, ^19^ ^]^ Furthermore, other mucosal tissues such as intestinal and ocular epithelia can also harbour HAdV,^[^ ^20^ ^]^ and handling of such tissue samples may also lead to contamination in laboratories. Infection of cell cultures with viruses is a huge problem due to difficulties in the detection and identification of these contaminants, given the symptomless effects at first sight. In addition, it is very difficult to eradicate viruses once cell cultures have established a persistent infection, as viruses do not respond to antibiotic treatment and are obligate parasites of the cell itself.^[^ ^21^ ^]^ HAdV is known for its high production of viral particles and efficient infection of host mammalian cells.^[^ ^22^ ^]^ Further considering that HAdVs remain clinically infectious and can be recovered on plastic and metal surfaces for up to a month,^[^ ^23^ ^]^ it is highly recommended to separate tonsillar cell and tissue cultures in a quarantine laboratory to prevent contamination of other cells cultures. In addition to cleaning with 70% ethanol, as recommended to eliminate the virus,^[^ ^24^ ^]^ formalin gas sterilization was employed for difficult to reach utilities (eg flow cabinet). For the rapid HAdV C detection of cell stocks, we developed an in‐house qPCR assay, and however, a wide variety of HAdV detection methods have previously been published and can be used as well.^[^ ^25^, ^26^, ^27^ ^]^ Since it is nearly impossible to remove viral contaminants from cell cultures, discarding valuable (patient‐derived) cell sources were inevitable.

The bacterial contamination with *Brevibacillus* sp that we encountered in our keratinocyte cell cultures was, in contrast to HAdV C, easier to identify because of the macroscopic appearance of the contaminated cultures. Again, 16S rRNA gene sequencing resulted in fast and successful determination. However, finding the source of this contamination appeared more problematic. Source identification of the demin water system explained the widespread contamination in our cell culture facility, as our 70% ethanol was prepared from demineralized water from the infected cartridges. Similar to an outbreak of Pseudobacteremia traced to ethanol‐resistant *Bacillus* spores present in the cotton ethanol swaps used to disinfect blood culture bottles,^[^ ^28^ ^]^ we unintendedly spread the *Brevibacillus* bacteria via our aseptic culturing techniques.

In conclusion, when cell cultures inexplicably die in absence of any bacterial or fungal infection, a viral contamination is suspected. Furthermore, universal 16S rRNA gene PCR primers, commonly used for next generation sequencing in the context of microbiome research, should be considered a valuable tool for determination strategies of (bacterial) contamination in cell cultures to facilitate disinfection. Although omitting the prophylactic use of antibiotics, at first, increased the contamination rate in our cell culture facility, the resulting improved aseptic cell culture techniques and the identification of unexpected sources of contamination positively influenced the quality of our research involving in vitro cell and tissue models.

## CONFLICT OF INTERESTS

The authors state no conflict of interests.

## AUTHOR CONTRIBUTIONS

EB and PZ supervised all laboratory experiments, interpreted the data and wrote the manuscript. HN performed keratinocyte cell cultures, bacterial growth experiments, interpreted the data and wrote the manuscript. J.Sc. has critically revised the manuscript. DR, GR and J.Sm. performed keratinocyte cell cultures, mouse fibroblasts cultures and human embryonic kidney cell cultures. PZ and PJ performed microbial DNA isolation, PCR amplification and sequencing. PZ developed HAdV C‐specific qPCR. D.R performed HAdV C qPCR on entire cell bank. All authors contributed to discussion and interpretation of the results and writing of the manuscript; all authors read and approved the manuscript.

## Supporting information


**Figure S1.** *Brevibacillus* sp contamination. Blood agar plates showing bacterial contamination of (A) demin tap water and (B) water from the water bath. (C) Demin tap water treated with chlorine solution (50 mg/L, pH 7.0).
**Data S1.** Methods.
