# Supplementary material for: Know your enemy: Unexpected, pervasive and persistent viral and bacterial contamination of primary cell cultures
Source: Exp Dermatol. 2020 Jun 25;29(7):672–6. doi: 10.1111/exd.14126 (PMC7496648; doi:10.1111/exd.14126)
Supplement: Supplementary file 1 — Figure S1. Brevibacillus sp contamination. Blood agar plates showing bacterial contamination of (A) demin tap water and (B) water from the water bath. (C) Demin tap water treated with chlorine solution (50 mg/L, pH 7.0). Data S1. Methods. [file EXD-29-672-s001.pdf]

Supplemental Information

Know your enemy: unexpected, pervasive and persistent viral and bacterial contamination of primary cell cultures

Niehues et al.

Supplemental figures

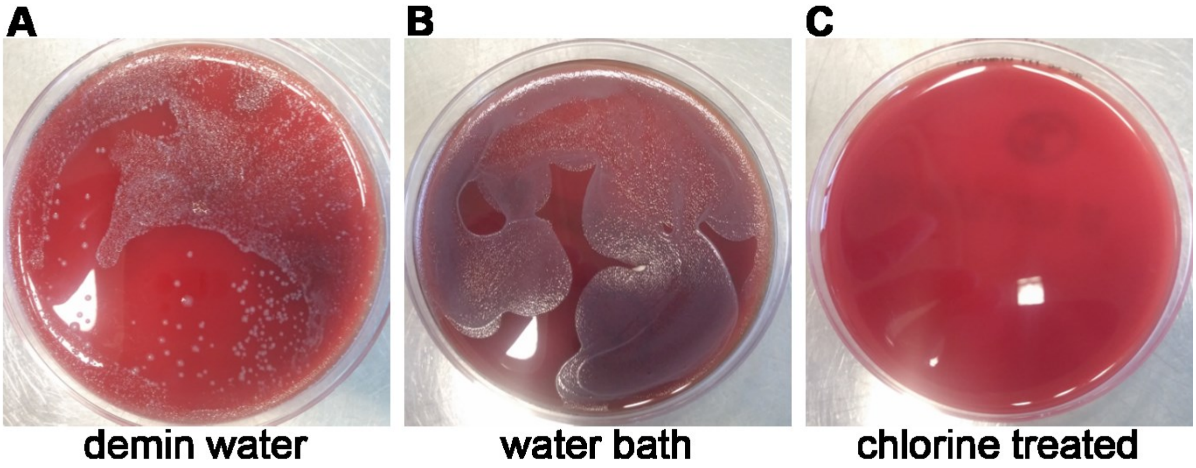

Figure S1. *Brevibacillus sp.* contamination. Blood agar plates showing bacterial contamination of (A) demin tap water and (B) water from the water bath. (C) Demin tap water treated with chlorine solution (50 mg/L, pH 7.0).

Data S1. Methods

Primary human keratinocyte cultures

Keratinocytes were isolated from human abdominal skin derived from donors who underwent surgery for abdominal wall correction, as previously described (1). All surgery procedures were performed after informed consent and in line with the principles and guidelines of the Declaration of Helsinki. Primary keratinocytes were cultured in keratinocyte growth medium (Lonza, Walkersville, MD, USA) and differentiated by depletion of growth factors, as described previously (2). Immortalized N/TERT keratinocytes (3) were cultured in K-SFM medium (ThermoFisher Scientific, Waltham, MA, USA).

Bacterial growth on blood agar plates

Cell culture supernatants and water bath and water tap samples were applied onto Columbia agar with 5% sheep blood (Becton, Dickinson and Co., Franklin Lakes, NJ, USA) and incubated both overnight and for 3 days at 37°C under both aerobic and anaerobic conditions.

Microbial DNA isolation, PCR amplification, and sequencing

Microbial gDNA was extracted using the MO BIO Ultraclean Microbial DNA Isolation Kit (MO BIO Laboratories, Carlsbad, AZ, USA) and universal primers were applied for amplification of the V3-V6 region of the 16S rRNA gene as described previously (4). The PCR amplicon of approximately 750 bp was subsequently purified using the GelElute DNA purification kit (Qiagen, Hilden, Germany) and submitted for Sanger sequencing at the Genomics Technology Center of the Radboudumc.

HAdV-C specific qPCR

Microbial gDNA was used as template for qPCR amplification with manually designed primers specific for HAdV-C (Human adenovirus C strain, complete genome, Genbank Accession: JX173086.1) and analysed using the MyiQ Single-Color Real-Time Detection System for quantification with SYBR Green and melting curve analysis (Bio-Rad Laboratories, Hercules, CA, USA). The HAdV-C specific primer pair (HAdV-C forward primer 5'-gtgcccattaagtaccgtgaatg-3' and HAdV-C reverse primer 5'- cagcgacagtgacactccagtag-3') is developed with freely available online Primer3 software package (http://bioinfo.ut.ee/primer3-0.4.0/) and primers were ordered at Biolegio, Nijmegen, The Netherlands.

Acknowledgements

Mycoplasma testing (Mycoalert™ detection kit, Lonza, France) was performed by Frank Oerlemans from the department of Cell Biology, Radboudumc.

Supplemental references

1. Rheinwald JG, Green H. Formation of a keratinizing epithelium in culture by a cloned cell line derived from a teratoma. Cell 1975: 6: 317-330.

2. van Ruissen F, de Jongh GJ, Zeeuwen PL, van Erp PE, Madsen P, Schalkwijk J. Induction of normal and psoriatic phenotypes in submerged keratinocyte cultures. J Cell Physiol 1996: 168: 442-452.

3. Smits JPH, Niehues H, Rikken G, et al. Immortalized N/TERT keratinocytes as an alternative cell source in 3D human epidermal models. Sci Rep 2017: 7: 11838.

4. Zeeuwen PL, Boekhorst J, van den Bogaard EH, et al. Microbiome dynamics of human epidermis following skin barrier disruption. Genome biology 2012: 13: R101.
